# Tongxinluo Capsule Combined with Atorvastatin for Coronary Heart Disease: A Systematic Review and Meta-Analysis

**DOI:** 10.1155/2021/9413704

**Published:** 2021-07-17

**Authors:** Qiao Liu, Taiwei Dong, Miaomiao Xi, Licheng Gou, Yang Bai, Lian Hou, Min Li, Li Ou, Feng Miao, Peifeng Wei

**Affiliations:** ^1^Shaanxi University of Chinese Medicine, Xianyang 712046, China; ^2^The Second Affiliated Hospital of Shaanxi University of Chinese Medicine, Xianyang 712000, China; ^3^Shaanxi Provincial Hospital of Traditional Chinese Medicine, Xi'an 710000, China

## Abstract

**Introduction:**

Coronary heart disease (CHD) is a common clinical cardiovascular disease, and its morbidity and mortality rates are increasing, which brings a serious burden to the family and society. Dyslipidemia is one of the most important risk factors for CHD. However, it is difficult to reduce blood lipids to an ideal state with the administration of a statin alone. Tongxinluo capsule (TXLC), as a Chinese patent medicine, has received extensive attention in the treatment of CHD in recent years. This systematic review and meta-analysis aim to provide evidence-based medicine for TXLC combined with atorvastatin in the treatment of CHD.

**Objective:**

To evaluate systematically the effectiveness and safety of TXLC combined with atorvastatin in the treatment of CHD.

**Methods:**

Seven English and Chinese electronic databases (PubMed, Cochrane Library, Embase, CNKI, VIP, CBM, and Wanfang) were searched from inception to January 2020, to search for randomized controlled trials (RCTs) on TXLC combined with atorvastatin in the treatment of CHD. Two researchers independently screened the literature according to the literature inclusion and exclusion criteria and performed quality assessment and data extraction on the included RCTs. We performed a systematic review following Cochrane Collaboration Handbook and Preferred Reporting Items for Systematic Reviews and Meta-Analyses (PRISMA) guidelines and using a measurement tool to assess the methodological quality of systematic reviews (AMSTAR 2). The quality of outcomes was evaluated by the Grading of Recommendations Assessment, Development and Evaluation (GRADE). And meta-analysis was performed by Review Manager 5.2.

**Results:**

A total of 15 RCTs with 1,578 participants were included in this review. Compared to atorvastatin treatment, TXLC combined with atorvastatin treatment showed potent efficacy when it came to the effectiveness of clinical treatment (RR = 1.24; 95% CI, 1.18, 1.29; *P* < 0.00001), total cholesterol (TC; MD = −1.21; 95% CI, −1.53, −0.89; *P* < 0.00001), triacylglycerol (TG; MD = −0.73; 95% CI, −0.81, −0.65; *P* < 0.00001), high-density lipoprotein cholesterol (HDL-C; MD = 0.27; 95% CI, 0.23, 0.31; *P* < 0.00001), low-density lipoprotein cholesterol (LDL-C; MD = –0.72; 95% CI, –0.80, −0.64; *P* < 0.00001), C-reactive protein (CRP; SMD = −2.06; 95% CI, −2.56, −1.57; *P* < 0.00001), frequency of angina pectoris (SMD = −1.41; 95% CI, −1.97, −0.85; *P* < 0.00001), duration of angina pectoris (MD = −2.30; 95% CI, −3.39, −1.21; *P* < 0.0001), and adverse reactions (RR = 0.84; 95% CI, 0.51, 1.39; *P*=0.50). No serious adverse events or reactions were mentioned in these RCTs. According to the PRISMA guidelines, although all studies were not fully reported in accordance with the checklist item, the reported items exceeded 80% of all items. With the AMSTAR 2 standard, the methodological quality assessment found that 9 studies were rated low quality and 6 studies were rated critically low quality. Based on the results of the systematic review, the GRADE system recommended ranking method was used to evaluate the quality of evidence and the recommendation level. The results showed that the level of evidence was low, and the recommendation intensity was a weak recommendation.

**Conclusions:**

TXLC combined with atorvastatin in the treatment of CHD can effectively improve the effectiveness of clinical treatment, significantly reduce the frequency and duration of angina pectoris, decrease blood lipids, and improve inflammatory factors. However, due to the low quality of the literature included in these studies and the variability of the evaluation methods of each study, there is still a need for a more high-quality, large sample, multicenter clinical randomized control for further demonstration.

## 1. Introduction

Coronary atherosclerotic heart disease, referred to as coronary heart disease (CHD), is caused by coronary atherosclerosis or spasm that causes the lumen to become blocked and narrow, blood flow to be blocked, and blood supply to become insufficient, which causes myocardial ischemia and no oxygen supply. A cardiovascular disease that causes necrosis is one of the diseases with the highest morbidity and mortality in the world [1, 2]. A large number of clinical studies have shown that the number of patients with CHD is increasing, and due to bad lifestyle habits such as smoking and drinking, the incidence trend is gradually showing younger age [3]. Patients mainly show symptoms such as angina pectoris and heart failure during attacks. In severe cases, they may even die suddenly [4], which greatly affects the daily life of patients and even threatens their lives. Therefore, safe and effective treatments are needed to prevent and treat this disease.

According to research, the incidence of cardiovascular disease can be reflected by blood lipid levels [5]. In patients with a high risk of CHD and similar critical conditions, low-density lipoprotein cholesterol (LDL-C) should be reduced by at least 30–50% in order to obtain a clinical benefit [6]. So the treatment of CHD can improve blood lipid levels by lipid-lowering drugs, effectively reduce LDL-C levels, and prevent the formation or development of coronary atherosclerotic plaque, thereby preventing the plaque from further rupture and thrombosis that may lead to myocardial infarction. Atorvastatin is commonly used clinically to treat CHD. It is a reductase inhibitor that can inhibit the synthesis of cholesterol, reduce TG levels, increase the activity of LDL receptors, and promote the metabolism of LDL-C [7]. The application of statins significantly reduced the incidence of adverse cardiovascular events and delayed the progression of coronary atherosclerosis [8]. However, it has been found in clinical practice that some patients with CHD still have adverse cardiovascular events after treatment with strong statins [9, 10]. CHD belongs to the category of chest obstruction, palpitations, and heartache in traditional Chinese medicine (TCM), and the clinical practice of Chinese medicine has confirmed that blood stasis is the core pathogenesis of chest obstruction [11]. Studies have shown that on the basis of statin therapy, the addition of TCM for promoting blood circulation and removing blood stasis can improve the efficacy of CHD [12]. Tongxinluo capsule (TXLC) is a kind of TCM preparation, mainly composed of ginseng, leech, whole scorpion, centipede, chuanxiong, and borneol, which has the effects of promoting blood circulation and removing blood stasis [13]. Modern pharmacological studies have shown that TXLC can lower cholesterol, improve microcirculation, relieve atherosclerosis, stabilize plaque, and inhibit inflammatory cell infiltration [14]. In recent years, it is used to treat CHD and has achieved good results. This study will perform a meta-analysis of the randomized controlled trials (RCTs) of TXLC combined with atorvastatin in the treatment of CHD and appropriately refers to relevant literature methods [15], in order to evaluate the efficacy and safety of the two drugs in the treatment of CHD.

## 2. Methods

### 2.1. Search Strategy

Comprehensive searches were conducted in both English and Chinese databases to identify all published RCTs from inception to January 2020. All relevant RCTs were searched from the following 7 databases including PubMed, Cochrane Library, Embase, CNKI, VIP, CBM, and Wanfang. The following search terms were used: “tongxinluo capsules” (title/abstract), AND “atorvastatin” (title/abstract), AND “cardiovascular disease” (title/abstract), OR “coronary heart disease” (title/abstract), OR “coronary artery disease” (title/abstract), OR “acute coronary syndrome” (title/abstract).

### 2.2. Inclusion Criteria

Two authors (Qiao Liu and Taiwei Dong) read the titles and abstracts of trials in all searched databases independently to assess the rationality for inclusion. The full text was further read to evaluate for the inclusion criteria. The inclusion criteria were as follows. (1) Diagnostic criteria for CHD: 1979 “Nomenclature and diagnostic criteria for ischemic heart disease” developed by World Health Organization (WHO). (2) Research protocol: the treatment group received TXLC and atorvastatin treatment, and the control group received atorvastatin or conventional and atorvastatin treatments. (3) Outcome indicators: the effectiveness of clinical treatment is the proportion of the total number of people who are significantly effective and effective. According to the WHO standard of CHD curative effect, it is divided as follows: significantly effective: angina pectoris symptoms disappeared significantly, and the electrocardiogram returned to normal; effective: angina pectoris symptoms were reduced to a certain extent, and the electrocardiogram was improved; and invalid: angina pectoris symptoms did not change, and the electrocardiogram did not change. Blood lipid levels include total cholesterol (TC), triglycerides (TG), low-degree lipoprotein cholesterol (LDL-C), high-density lipoprotein cholesterol (HDL-C), inflammatory factors including C-reactive protein (CRP), and adverse reactions. (4) Research type: randomized controlled trials (RCTs).

### 2.3. Exclusion Criteria

The trials conforming to the following conditions were excluded: (1) noncardiogenic chest pain; (2) cases included in the study that included myocardial infarction, rheumatic heart disease, cardiomyopathy, severe heart rhythm disorders, severe heart failure, and so on; (3) reduplicative publications reporting the same trials; (4) nonrandomized controlled trials; (5) nonclinical experiments, reviews, literature research, mechanism research, or animal experiment; (6) controlled interventions combined with any other medicine in the control or treatment group; (7) incorrect data for meta-analysis; (8) patients with unclear functional classification; and (9) trials with unclear evaluation indicators or basic data for statistic research.

### 2.4. Reporting Quality of Included RCTs

The Preferred Reporting Items for Systematic Reviews and Meta-Analyses (PRISMA) statement [16] is composed of a 27-item checklist and a 4-phase flow diagram. The 7 parts of the checklist item are the title, abstract, introduction, methods, results, discussion, and funding. Each item has options such as “yes,” “no,” or “not applicable”. The flow diagram is composed of identification, screening, eligibility, and included.

### 2.5. Data Extraction

Based on the search strategy, two investigators (Taiwei Dong and Miaomiao Xi) combined the literature inclusion and exclusion criteria, independently screened the literature, excluded the irrelevant literature, and checked it. When there was a disagreement, the third researcher (Feng Miao) participated in the discussion and jointly evaluated the basic content including the first author and publication year; random method; number of cases in the treatment and control groups; age; gender; specific intervention measures and course of treatment; and outcome indicators.

### 2.6. Evaluation of Literature Quality

#### 2.6.1. Assessment of Risk of Bias

Two researchers (Qiao Liu and Taiwei Dong) independently assessed the included RCTs based on the bias risk assessment criteria recommended by the Cochrane Collaboration Handbook. The methodological criteria and methods of evaluation are as follows: (1) random sequence generation, (2) allocation concealment, (3) blinding of participants and personnel, (4) blinding of outcome assessment, (5) incomplete outcome data, (6) selective reporting, and (7) other bias. We conduct bias risk assessments for each RCT and classify them as “high risk,” “uncertain risk,” or “low risk.” Two researchers discussed according to the above criteria and methods, and if necessary, they could intervene through a third evaluator (Peifeng Wei) to negotiate and finally reach a consensus.

#### 2.6.2. Quality Assessment of Systematic Reviews

The methodological quality of all included RCTs was assessed using Assessing the Methodological Quality of Systematic Reviews (AMSTAR 2) [17]. The domain-specific questions in AMSTAR 2 are framed so that a “yes” answer denotes a positive result. If no information is provided to rate an item, the item should be rated as a “no.” We can choose a “partial yes” response in some instances where we considered it worthwhile to identify partial adherence to the standard. A detailed description of AMSTAR 2 is provided in Table 1. Based on the information provided by each RCT, two researchers conducted methodological quality assessments through AMSTAR 2. If there is a disagreement, the third researcher can negotiate a settlement.

#### 2.6.3. Evidence Quality and Recommendation Level

Based on the results of systematic reviews, the GRADE system recommended ranking method [33] was used to evaluate the quality of evidence and the recommendation level. GRADE evidence quality assessment can divide the importance of the assessment results into 3 levels, of which 1–3 are unimportant outcome indicators, 4–6 are important outcome indicators, and 7–9 are critical outcome indicators. Since the treatment of CHD is mainly to improve the patient's condition by reducing blood lipids, 5 indicators of clinical treatment effectiveness, TC, TG, HDL-C, and LDL-C are used as critical outcome indicators. CRP, frequency of angina pectoris, duration of angina pectoris, and adverse reactions were used as important outcome indicators. RCTs were initially defined as high-quality evidence, and observational studies are defined as low-quality evidence. According to the research design, the further evidence on increase and decrease factors are determined by 5 downgrade factors and 4 upgrade factors [34]. The recommendation level is divided into “strong recommendation” and “weak recommendation”: strong recommendation indicates that the evaluator is convinced that the intervention has more advantages than disadvantages or disadvantages than benefits, and weak recommendation indicates that the advantages and disadvantages are uncertain. Finally, GRADEpro 3.6 software was used to analyze and chart the quality of evidence, and the recommended level was given based on the quality of evidence combined with the research theme. The evaluation of the promotion and demotion factors is the responsibility of QiaoLiu and Miaomiao Xi. If there is a dispute, the third researcher (Peifeng Wei) is required to review and reach an agreement through discussion.

### 2.7. Statistical Analysis of Data

Reviewer Manager 5.2 software provided by Cochrane was used for meta-analysis of the literature. For outcome measures, dichotomous variables were presented as risk ratio (RR) with 95% confidence intervals (CI), while continuous outcomes were expressed as mean difference (MD) with 95% CI; if each trial data uses different measurement tools and different measurement units to record data, the standardized mean difference (SMD) is used for analysis. As a quantitative measure of inconsistency, the *I*-square (*I*^2^) statistic was used to assess heterogeneity. The fixed effects model was performed with minor heterogeneity when *I*^2^ was less than 50%. The random effects model was applied when *I*^2^ was over 50%. Meanwhile, a funnel plot was used for assessing the potential publication bias. The data was entered by Qiao Liu and supervised by Taiwei Dong.

## 3. Results

### 3.1. Literature Screening Process and Results

**Figure 1:**
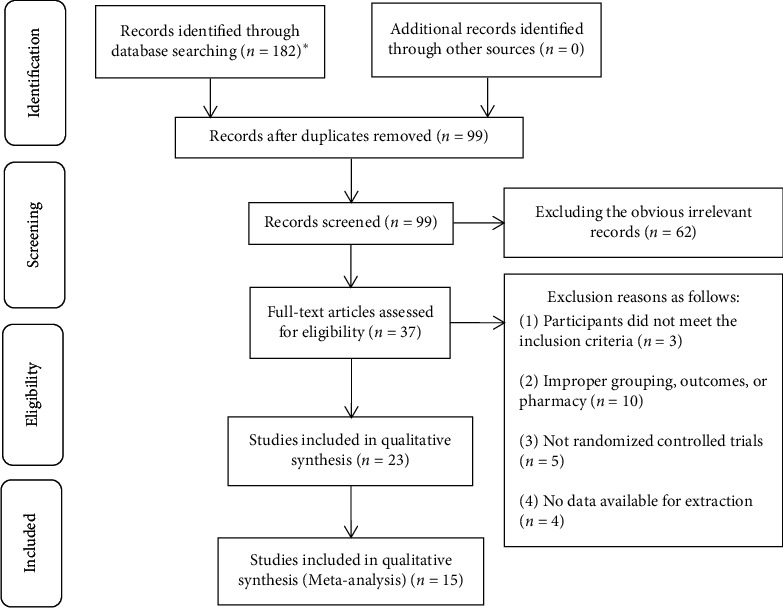
Literature search: PRISMA flow diagram. ^*∗*^The number of databases and documents retrieved are as follows: PubMed (*n* = 3), Cochrane Library (*n* = 0), Embase (*n* = 0), CNKI (*n* = 54), VIP (*n* = 39), CBM (*n* = 27), and Wanfang (*n* = 59).

The PRISMA flow diagram is presented in Figure 1. A total of 182 records were identified for preliminary screening after searching English and Chinese databases. All the included trials were conducted in China and published in Chinese. 99 records were reserved for further screening after removing 83 duplicated publications. For the preserved records, 62 obvious irrelevant literature were excluded by reading the title and abstract. 37 full-text articles were used for further assessment. After reading the full text, 22 more literature works were excluded for the following reasons: participants not meeting the inclusion criteria (*n* = 3); improper grouping, outcomes, or pharmacy (*n* = 10); nonrandomized controlled trials (*n* = 5); and no data available for extraction (*n* = 4). Finally, 15 RCTs of TXLC combined with atorvastatin for CHD were included in this review.

### 3.2. Study Characteristics

As shown in Table 2, a total of 15 RCTs with 1,578 participants were included in this review. The control group consisted of 789 patients, while the treatment group consisted of 789 patients. All trials' sample sizes ranged from 60 to 160. As for the characteristics of the intervention, the course of treatment varied from 4 weeks to 3 months. Only 1 trial did not mention the course of treatment [20]. The baseline of patients in both groups was balanced. The treatment group used TXLC combined with the same atorvastatin and conventional treatment as a control group, and 4 trials used only atorvastatin [18, 20, 21, 24]. Most trials in the treatment group used the dose of 12 capsules per day; 3 trials used 9 capsules per day; and only 1 trial used 6 to 12 capsules per day. TXLC was given through oral administration 3 times daily in all included trials. Most trials in the control group used atorvastatin dose of 20 mg/d; 2 trials used 10 mg/d; 2 trials used 1 tablet per day; and only 1 trial adjusted the dosage according to the specific conditions of the patient. The control group used conventional medical treatment, including nitrate drugs, aspirin antiplatelet therapy, heparin anticoagulant therapy, and *β*-receptor blockers. Fourteen trials reported the effectiveness of clinical treatment. Nine trials reported TC and TG. Eight trials reported LDL-C. Seven articles reported CRP. Six articles reported HDL-C and adverse reactions. Five trials reported the frequency of angina pectoris. Four trials reported the duration of angina pectoris.

### 3.3. Reporting Quality Results of Included RCTs

The included RCTs were not well reported due to the incomplete implementation of the PRISMA statement. None of the studies reported on protocol and registration; to avoid or minimize the risk of bias in a study [29], six studies [19–21, 29, 31, 32] were not subjected to additional analysis, and the remaining projects were fully reported. PRISMA's checklist item is shown in Table 3.

### 3.4. Quality Assessment of Systematic Reviews

According to the AMSTAR 2 standard for methodological quality evaluation, 9 studies were rated as “low”, and 6 studies were rated as “critically low.” None of the studies mentioned that the review method was established before the review was conducted, and none of the studies reported the source of funding; 6 studies [19–21, 29, 31, 32] failed to combine the results statistically; 1 study [29] did not assess the risk of bias; 4 studies [19, 21, 31, 32] failed to provide a satisfactory explanation for the existence of heterogeneity; and 1 study [21] failed to adequately investigate publication bias (Table 1).

### 3.5. Risk of Bias Assessment in Included RCTs

**Figure 2:**
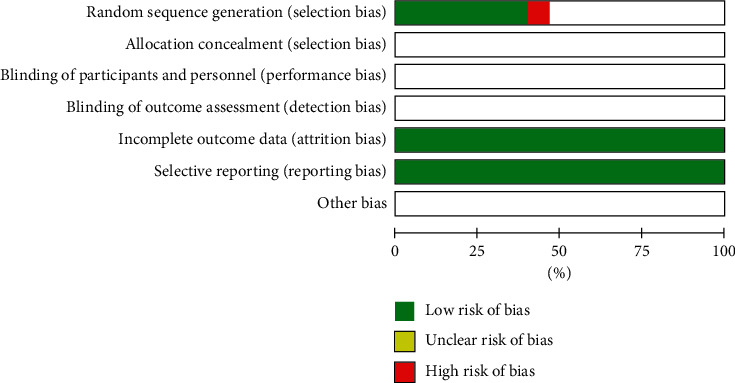
Risk of bias assessment of included studies.

Six of the included RCTs [18, 21, 23, 25, 27, 30] used the random number table method for allocation, and the remaining five trials [19, 20, 22, 24, 26] only mentioned random allocation, but there is no specific description of the random method, Only one reported that random sequence was parity of hospital order [29]. All trials do not mention whether to use allocation hiding and whether to blind doctors and patients. No subjects who dropped out. And other potential sources of bias are unclear as shown in Figure 2.

AMSTAR 2 checklist is as follows:

1. Did the research questions and inclusion criteria for the review include the components of PICO?
2. Did the report of the review contain an explicit statement that the review methods were established prior to the conduct of the review, and did the report justify any significant deviations from the protocol?
3. Did the review authors explain their selection of the study designs for inclusion in the review?
4. Did the review authors use a comprehensive literature search strategy?
5. Did the review authors perform study selection in duplicate?
6. Did the review authors perform data extraction in duplicate?
7. Did the review authors provide a list of excluded studies and justify the exclusions?
8. Did the review authors describe the included studies in adequate detail?
9. Did the review authors use a satisfactory technique for assessing the risk of bias (RoB) in individual studies that were included in the review?
10. Did the review authors report on the sources of funding for the studies included in the review?
11. If meta-analysis was performed, did the review authors use appropriate methods for statistical combination of results?
12. If meta-analysis was performed, did the review authors assess the potential impact of RoB in individual studies on the results of the meta-analysis or other evidence synthesis?
13. Did the review authors account for RoB in individual studies when interpreting/discussing the results of the review?
14. Did the review authors provide a satisfactory explanation for, and discussion of, any heterogeneity observed in the results of the review?
15. If they performed quantitative synthesis, did the review authors carry out an adequate investigation of publication bias (small study bias) and discuss its likely impact on the results of the review?
16. Did the review authors report any potential sources of conflict of interest, including any funding they received for conducting the review?

### 3.6. Meta-Analysis Results

#### 3.6.1. Meta-Analysis Based on the Effectiveness of Clinical Treatment

**Figure 3:**
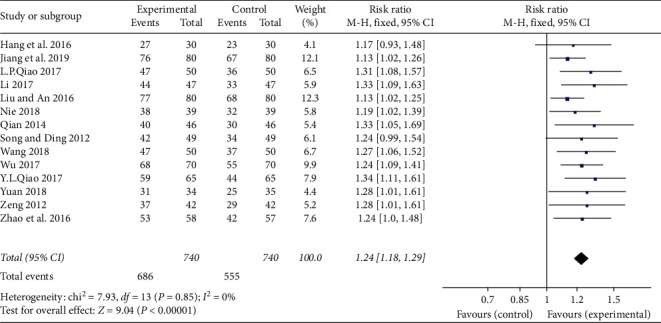
Forest plot of TXLC plus atorvastatin versus atorvastatin on the effectiveness of clinical treatment in patients with CHD.

A total of 14 trials with 1,480 patients investigated the effectiveness of clinical treatment of TXLC plus atorvastatin versus atorvastatin in patients with CHD [18–29, 31, 32]. There were 740 patients in the treatment group and 740 in the control group. The results showed that there was no heterogeneity (*P*=0.85; *I*^2^ = 0%), and the fixed effects model was adopted for analysis. As shown in the forest plot, there was a statistically significant difference between TXLC plus atorvastatin and atorvastatin in the effectiveness of clinical treatment (RR = 1.24; 95% CI, 1.18, 1.29; *P* < 0.00001; Figure 3).

#### 3.6.2. Meta-Analysis Based on Total Cholesterol (TC)

**Figure 4:**
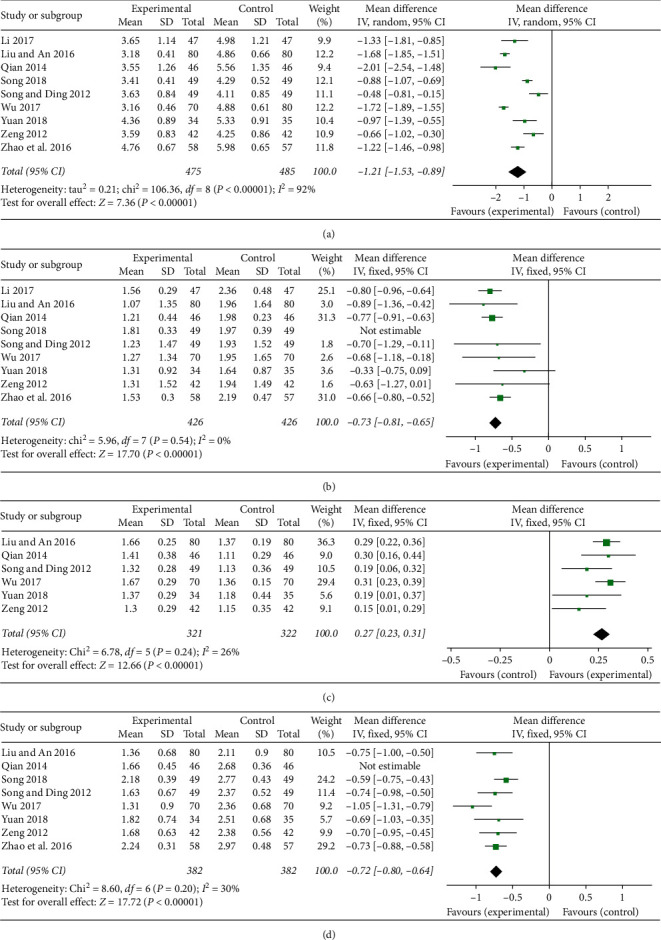
Forest plot of TXLC plus atorvastatin versus atorvastatin in decreasing TC, TG, and LDL-C and in increasing HDL-C: (A) the forest plot of TC, (B) the forest plot of TG, (C) the forest plot of HDL-C, and (D) the forest plot of LDL-C.

Nine trials with 960 participants assessed the effect of TXLC plus atorvastatin versus atorvastatin in decreasing TC in patients with CHD [19, 21, 23–26, 29, 31, 32]. It has considerably high heterogeneity in TC (*P* < 0.00001; *I*^2^ = 92%), and the random effects model was used to combine effect quantities for analysis. The results showed that TXLC plus atorvastatin was superior to atorvastatin treatment to reduce TC; the difference was statistically significant (MD = -1.21; 95% CI, −1.53, −0.89; *P* < 0.00001; Figure 4(a)). Due to the limited number of studies, funnel plot analysis was not available. Sensitivity analysis found that excluding 9 studies, the combined effects were still statistically significant, and the direction of the forest plot results did not change.

#### 3.6.3. Meta-Analysis Based on Triacylglycerol (TG)

Nine trials assessed the effect of TXLC plus atorvastatin versus atorvastatin in decreasing TG in patients with CHD [19, 21, 23–26, 29, 31, 32]. It has considerably high heterogeneity in TG (*P* < 0.00001; *I*^2^ = 84%); after excluding studies that may cause heterogeneity [21], the heterogeneity disappeared (*P*=0.54; *I*^2^ = 0%), so a fixed effects model was conducted for analysis. The results showed that TXLC plus atorvastatin could substantially reduce the level of TG compared with atorvastatin treatment (MD = −0.73; 95% CI, −0.81, −0.65; *P* < 0.00001; Figure 4(b)). Due to the limited number of studies, funnel plot analysis was not available.

#### 3.6.4. Meta-Analysis Based on High-Density Lipoprotein Cholesterol (HDL-C)

A total of 6 trials with 643 patients evaluated HDL-C and were pooled with a fixed model [19, 24, 26, 29, 31, 32]. The heterogeneity of the HDL-C study was considerably low (*P*=0.24; *I*^*2*^ = 26%). The results showed that TXLC plus atorvastatin was superior to atorvastatin treatment to increase HDL-C; the difference was statistically significant (MD = 0.27; 95% CI, 0.23, 0.31; *P* < 0.00001; Figure 4(c)). Due to the limited number of studies, funnel plot analysis was not available.

#### 3.6.5. Meta-Analysis Based on Low-Density Lipoprotein Cholesterol (LDL-C)

Eight trials assessed the therapy of TXLC plus atorvastatin versus atorvastatin in decreasing LDL-C in patients with CHD [19, 21, 24–26, 29, 31, 32]. It has considerably high heterogeneity in LDL-C (*P* < 0.010; *I*^2^ = 62%); after excluding studies that may cause heterogeneity [26], the heterogeneity reduced (*P*=0.20; *I*^2^ = 30%), so a fixed effects model was conducted for analysis. The results showed that TXLC plus atorvastatin was superior to atorvastatin treatment to reduce LDL-C; the difference was statistically significant (MD = −0.72; 95% CI, −0.80, −0.64; *P* < 0.00001; Figure 4(d)). Due to the limited number of studies, funnel plot analysis was not available.

#### 3.6.6. Meta-Analysis Based on C-Reactive Protein (CRP)

A total of 7 trials with 690 patients evaluated CRP and were pooled with a random model [18, 23–28]. The heterogeneity was considerably high (*P* < 0.00001; *I*^2^ = 85%). Due to the different measurement units used in CRP in various studies, we use SMD as the effect indicator for meta-analysis. The results showed that TXLC plus atorvastatin was superior to atorvastatin treatment to reduce CRP; the difference was statistically significant (SMD = −2.06; 95% CI, −2.56, −1.57; *P* < 0.00001; Table 4). Due to the limited number of studies, funnel plot analysis was not available. Sensitivity analysis found that excluding 7 studies, the combined effects were still statistically significant, and the direction of the forest plot results did not change.

#### 3.6.7. Meta-Analysis Based on Frequency of Angina Pectoris

Five trials with 532 participants assessed the effect of TXLC plus atorvastatin versus atorvastatin in decreasing the frequency of angina pectoris in patients with CHD and were pooled with a random model [22, 23, 27, 28, 31]. It has considerably high heterogeneity in the frequency of angina pectoris (*P* < 0.00001; *I*^2^ = 88%). Due to the different measurement units used for the frequency of angina pectoris in various studies, we use SMD as an effective indicator for meta-analysis. The results showed that TXLC plus atorvastatin was superior to atorvastatin treatment to reduce the frequency of angina pectoris; the difference was statistically significant (SMD = −1.41; 95% CI, −1.97, −0.85; *P* < 0.00001; Table 4). Due to the limited number of studies, funnel plot analysis was not available.

#### 3.6.8. Meta-Analysis Based on Duration of Angina Pectoris

Four trials with 372 participants assessed the effect of TXLC plus atorvastatin versus atorvastatin in decreasing the duration of angina pectoris [22, 23, 27, 30]. There was considerable heterogeneity in the duration of angina pectoris (*P* < 0.00001, *I*^2^ = 93%) in trials. Meta-analysis with a random effects model showed that compared with atorvastatin treatment, TXLC plus atorvastatin significantly improves angina symptoms. The pooled analysis indicated that there was a statistically significant difference between TXLC plus atorvastatin and atorvastatin treatment to reduce the duration of angina pectoris (MD = −2.30; 95% CI, −3.39, −1.21; *P* < 0.0001; Table 4). Due to the limited number of studies, funnel plot analysis was not available. Sensitivity analysis found that excluding 4 studies, the combined effects were still statistically significant, and the direction of the forest plot results did not change.

#### 3.6.9. Meta-Analysis of Adverse Reactions

Six trials with 695 participants assessed the effect of TXLC plus atorvastatin versus atorvastatin in adverse reactions [19–21, 24, 29, 31]. The results showed that there was no heterogeneity (*P*=0.92; *I*^2^ = 0%). Meta-analysis with a fixed effects model showed that compared with atorvastatin treatment, TXLC plus atorvastatin significantly improves angina symptoms. The pooled analysis indicated that there was no significant difference between TXLC plus atorvastatin and atorvastatin treatment on adverse reactions (RR = 0.84; 95% CI, 0.51, 1.39; *P*=0.50; Table 4). Due to the limited number of studies, funnel plot analysis was not available.

### 3.7. Subgroup Analysis

**Figure 5:**
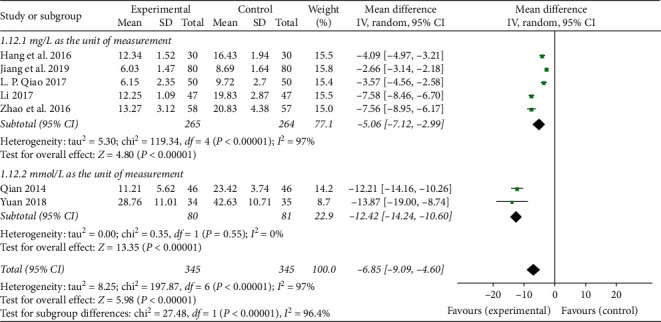
Forest plot of subgroup analysis on the CRP of TXLC plus atorvastatin versus atorvastatin in the treatment of CHD.

**Figure 6:**
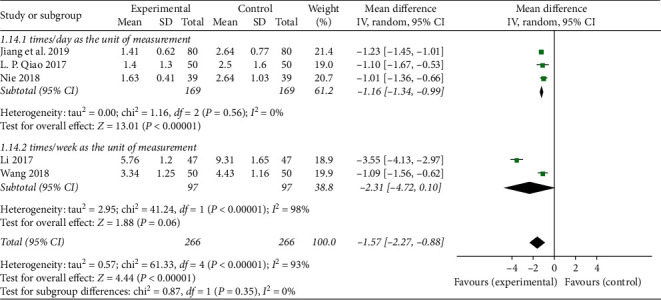
Forest plot of subgroup analysis on the frequency of angina pectoris of TXLC plus atorvastatin versus atorvastatin in the treatment of CHD.

Through the meta-analysis of CRP and the frequency of angina pectoris, we believe that the reason for the high heterogeneity may be due to the different measurement units used in the two outcome indicators in each study. Therefore, subgroup analysis of CRP and frequency of angina pectoris is performed according to the different measurement units. In the subgroup analysis of CRP, 5 studies [18, 23, 25, 27, 28] used “mg/L” as the measurement unit, and 2 studies [24, 26] used “mmol/L” as the measurement unit. The results showed that the subgroup with the measurement unit “mg/L” still has heterogeneity (*P* < 0.00001; *I*^2^ = 97%; Figure 5), and the subgroup with the unit of measurement “mmol/L” has no heterogeneity (*P*=0.55; *I*^2^ = 0%; Figure 5), indicating that the heterogeneity is affected by the inconsistent measurement unit, but there may be other factors. In the subgroup analysis of the frequency of angina pectoris, 3 studies [22, 27, 28] used “times/day” as the measurement unit, and 2 studies [23, 30] used “times/week” as the measurement unit, and the results showed that the subgroup with the measurement unit “times/day” has no heterogeneity (*P*=0.56; *I*^2^ = 0%; Figure 6), and the subgroup with the measurement unit “time/week” has heterogeneity (*P* < 0.00001; *I*^2^ = 98%; Figure 6), indicating that the heterogeneity is affected by the inconsistency of the measurement unit, but there may still be other factors.

### 3.8. Publication Bias

Publication bias was assessed using a funnel plot based on the effectiveness of clinical treatment reported in 14 studies. The funnel plot was asymmetrical (Figure S1). Furthermore, through Egger's test *P*=0.001 (Figure S2), the results showed that there was publication bias among the studies. And the bias might result from these reasons: small sample size, poor quality, and a high proportion of positive results.

### 3.9. GRADE Evidence Quality

This study has 9 outcome indicators, which are 5 critical outcome indicators: effectiveness of clinical treatment, TC, TG, HDL-C, LDL-C and 4 important outcome indicators: frequency of angina pectoris, duration of angina pectoris, CRP, and adverse reactions; the GRADE system evidence level of each outcome and the reasons for the promotion and demotion are shown in Table 5.

## 4. Discussion

### 4.1. Evaluation of Clinical Effectiveness and Safety

CHD belongs to the category of “thoracodynia” in TCM [35]. The occurrence of the disease is related to poor blood circulation in the body, and it leads to stasis of qi and blood and damaged blood vessels and further causes various symptoms such as paroxysmal chest pain [36]. Various factors such as overwork, emotional stress, stress, circulation factors, and so on may cause the onset of this symptom [37], and the sustained development of angina may lead to interruption of blood supply and myocardial infarction. Its main pathogenesis includes coronary artery lipid deposition, atherosclerotic plaque formation, and disorders of lipid metabolism. Therefore, the key to the treatment of CHD is to effectively adjust the blood lipid concentration, improve the tolerance of myocardial cells to ischemia, and improve the blood hypercoagulability state while improving myocardial blood supply [38]. TXLC has the effect of nourishing qi and activating blood, dredge meridians, and pain relief. Basic research proves that TXLC can promote the improvement of microcirculation, relieve atherosclerosis, and has the effect of inhibiting the inflammatory response and thrombosis [39–41]. The active ingredients in TXLC can promote the reduction of lipid deposition in plaques and have an inhibitory effect on the infiltration of inflammatory cells [42, 43], so they can be used for the treatment of cardiovascular and cerebrovascular diseases and relieve angina [44, 45]. Atorvastatin calcium is a selective and competitive inhibitor of HMG-CoA reductase [46]. The drug has the effects of regulating lipids, inhibiting endogenous cholesterol synthesis, and reducing inflammatory cytokine levels [47–49]. Recent studies showed that atorvastatin combined with TXLC can further reduce blood lipid levels, while the incidence of adverse events did not increase [50]. The combined use of the two not only produced beneficial effects on blood lipids but also effectively relieved myocardial ischemia, inhibited inflammation, and resisted atherosclerosis [51].

This study conducted a systematic evaluation according to the PRISMA guidelines and AMSTAR 2 standards. The results showed that the report of the systematic evaluation was not sufficient, and the methodological quality evaluation of all RCTs was of low or critically low quality, suggesting that the quality of the systematic review needs to be further improved. Through a comprehensive analysis of the outcome indicators of the included 15 studies, the results of the meta-analysis showed that TXLC combined with the atorvastatin group was significantly better than the atorvastatin group in terms of clinical treatment effectiveness. In terms of blood lipid levels, the treatment group can lower the levels of TC, TG, and LDL-C and increase the level of HDL-C better than the control group. In terms of inflammatory factors, the treatment group can reduce the level of CRP more than the control group. However, the heterogeneity among the researches of various indicators is large, suggesting poor stability. For the CRP and frequency of angina pectoris, due to the different measurement units between the studies, the subgroup analysis of the CRP and frequency of angina pectoris based on the measurement unit shows that the heterogeneity is affected by the measurement unit to some extent, but it still exists other influencing factors. A total of 6 studies in the included studies reported adverse events, mainly including gastrointestinal reactions such as nausea, vomiting, loss of appetite, muscle aches, abnormal liver function, and so on. No serious adverse reactions occurred. And the results showed that there was no statistical significance between the two groups, suggesting that the safety effect may not be obvious. At the same time, since most studies did not mention adverse events, it is recommended to increase the importance of drug safety in the future and improve the observation and reporting of safety indicators to increase the clinical reference value of the research.

### 4.2. Limitations of This Study

The study implemented strict inclusion and exclusion criteria. However, as the meta-analysis is a secondary study, it also has certain limitations, mainly considering factors, limited sample sizes, and changes in treatment time. High heterogeneity still exists among some of the outcome indicators. The following five issues remain in all RCTs from the results: (1) The amount of included trials is small, in addition to the lack of high-quality and large sample study. (2) Quality is generally low; the random application is less; and blind implementation is unknown. (3) Partial outcome indicators are subject to publication bias. Although extensive search strategies are used, supplements such as supplements, conference papers, and some gray literature are not available, and the inclusion of research information is limited. The study can only evaluate relevant indicators and cannot eliminate potential publication bias. (4) Languages, regions, and so on are also an issue. Although the language search is not restricted in this research, 15 articles were included in the Chinese literature and 0 articles in English after the search, which may affect the extrapolation of the research results. (5) Most of the studies did not report adverse reactions.

### 4.3. GRADE Systematic Evaluation of Evidence Quality and Recommendation Grade

In the GRADE system, although the evidence based on RCTs was initially rated as high quality, our confidence in this type of evidence may be reduced by five points. According to the GRADE methodology quality evaluation, two outcome indicators were rated as moderate; six outcome indicators were rated as low; and one outcome indicator was rated as very low for the following reasons. (1) Hidden and blind methods are missing. Therefore, there are research limitations. (2) Unit conversion may lead to heterogeneity between studies. (3) Some studies include fewer patients and observations, and the results are inaccurate. (4) There was publication bias. At the same time, the critical outcome indicators of this study are the effectiveness of clinical treatment and blood lipid indicators (TC, TG, LDL-C, and HDL-C), and the quality of evidence is generally low. And in view of the low quality of included RCTs, the authenticity of the conclusions was affected. These studies are all domestic and affect the extrapolation of conclusions, so the recommendation level is a weak recommendation.

## 5. Conclusions

In summary, the results of systematic review and meta-analysis suggest that TXLC combined with atorvastatin would benefit patients with CHD. However, based on the GRADE system, the recommendation level is a weak recommendation, and the quality of articles is low; more accurate conclusions may need to be collected more new research data, a full review of other language literature, and so on. At the same time, a more rigorous trial design is indispensable, especially the need for large-scale, multicenter, randomized, randomized, and double-blind RCTs. It is recommended to design large samples and high-quality research and adopt key indicators in strict accordance with the consolidated standards of reporting trials (CONSORT) standard [52] for further demonstrations, so as to draw more reliable conclusions to guide clinical practice.

## Figures and Tables

**Table 1:** AMSTAR 2 assessment for included meta-analyses.

Authors and year of included studies	AMSTAR 2 item																
	1	2^*∗*^	3	4^*∗*^	5	6	7^*∗*^	8	9^*∗*^	10	11^*∗*^	12	13^*∗*^	14	15^*∗*^	16	Ranking of quality
Hang et al. 2016 [18]	Yes	No	Yes	PY	Yes	Yes	PY	PY	PY	No	Yes	Yes	Yes	Yes	Yes	Yes	Low
Liu and An 2016 [19]	Yes	No	Yes	PY	Yes	Yes	PY	PY	PY	No	No	Yes	Yes	No	Yes	Yes	Critically low
Qiao 2017 [20]	Yes	No	Yes	PY	Yes	Yes	PY	PY	PY	No	No	Yes	Yes	Yes	Yes	Yes	Critically low
Song 2018 [21]	Yes	No	Yes	PY	Yes	Yes	PY	PY	PY	No	No	Yes	Yes	No	No	Yes	Critically low
Nie 2018 [22]	Yes	No	Yes	PY	Yes	Yes	PY	PY	PY	No	Yes	Yes	Yes	Yes	Yes	Yes	Low
Li 2017 [23]	Yes	No	Yes	PY	Yes	Yes	PY	PY	PY	No	Yes	Yes	Yes	Yes	Yes	Yes	Low
Yuan 2018 [24]	Yes	No	Yes	PY	Yes	Yes	PY	PY	PY	No	Yes	Yes	Yes	Yes	Yes	Yes	Low
Zhao et al. 2016 [25]	Yes	No	Yes	PY	Yes	Yes	PY	PY	PY	No	Yes	Yes	Yes	Yes	Yes	Yes	Low
Qian 2014 [26]	Yes	No	Yes	PY	Yes	Yes	PY	PY	PY	No	Yes	Yes	Yes	Yes	Yes	Yes	Low
Qiao 2017 [27]	Yes	No	Yes	PY	Yes	Yes	PY	PY	PY	No	Yes	Yes	Yes	Yes	Yes	Yes	Low
Jiang et al. 2019 [28]	Yes	No	Yes	PY	Yes	Yes	PY	PY	PY	No	Yes	Yes	Yes	Yes	Yes	Yes	Low
Wu 2017 [29]	Yes	No	Yes	PY	Yes	Yes	PY	PY	PY	No	No	No	No	No	Yes	Yes	Critically low
Wang 2018 [30]	Yes	No	Yes	PY	Yes	Yes	PY	PY	PY	No	Yes	Yes	Yes	Yes	Yes	Yes	Low
Song and Ding 2012 [31]	Yes	No	Yes	PY	Yes	Yes	PY	PY	PY	No	No	Yes	Yes	No	Yes	Yes	Critically low
Zeng 2012 [32]	Yes	No	Yes	PY	Yes	Yes	PY	PY	PY	No	No	Yes	Yes	No	Yes	Yes	Critically low
No. of yes	15	0	15	0	15	15	0	0	0	0	9	14	14	10	14	15	

^*∗*^The critical items of the AMSTAR 2; PY: partial yes; high: no or one noncritical weakness; moderate: more than one noncritical weakness; low: one critical flaw with or without noncritical weaknesses; and critically low: more than one critical flaw with or without noncritical weaknesses.

**Table 2:** Principal characteristics of the studies included in the meta-analysis.

Included research and year	Stochastic method	Sample size (*n*)		Age (y)		Male (%)		Intervening measure		Duration	Outcome measures
		*T*	*C*	*T*	*C*	*T*	*C*	*T*	*C*		
Hang et al. 2016 [18]	Random number table	30	30	62.45 (7.12)	62.62 (7.48)	57%	57%	C + TXLC 2–4 capsules each time, tid	Atorvastatin 20 mg/d	8 W	①⑧
Liu and An 2016 [19]	NR	80	80	54.5 (2.1)	51.1 (1.4)	61%	65%	C + TXLC 4 capsules each time, tid	CT + atorvastatin 20 mg/d	3 M	①④⑤⑥⑦⑨
Qiao [20]	NR	65	65	59.1 (6.5)	58.8 (7.1)	51%	51%	C + TXLC 2 capsules each time, tid	Atorvastatin 20 mg/d	NR	①⑨
Song [21]	Random number table	49	49	65.31 (10.42)	64.97 (9.55)	53%	51%	C + TXLC 3 capsules each time, tid	Atorvastatin 20 mg/d	3 M	④⑤⑥⑨
Nie [22]	NR	39	39	55.31 (2.6)	54.35 (2.17)	56%	54%	C + TXLC 4 capsules each time, tid	CT + atorvastatin 1 tablet/d	3 M	①②③
Li [23]	Random number table	47	47	61.87 (5.31)	62.34 (4.97)	57%	55%	C + TXLC 3 capsules each time, tid	CT + atorvastatin 20 mg/d	8 W	①②③④⑤⑧
Yuan 2018 [24]	NR	34	35	51.7 (3.2)	51.7 (3.2)	54%	54%	C + TXLC 3 capsules each time, tid	Atorvastatin 10 mg/d	4 W	①④⑤⑥⑦⑧⑨
Zhao et al. 2016 [25]	Random number table	58	57	59.8 (7.6)	59.0 (7.4)	59%	61%	C + TXLC 4 capsules each time, tid	CT + atorvastatin 10 mg/d	4 W	①④⑤⑥⑧
Qian 2014 [26]	NR	46	46	64.91 (4.38)	66.01 (4.45)	52%	59%	C + TXLC 4 capsules each time, tid	CT + atorvastatin 20 mg/d	3 M	①④⑤⑥⑦⑧
Qiao 2017 [27]	Random number table	50	50	64.2 (4.9)	65.1 (5.3)	62%	58%	C + TXLC 4 capsules each time, tid	CT + atorvastatin 20 mg/d	2 M	①②③⑧
Jiang et al. 2019 [28]	NR	80	80	58.5 (6.4)	59.1 (6.2)	58%	55%	C + TXLC 4 capsules each time, tid	CT + atorvastatin 20 mg/d	3 M	①②⑧
Wu 2017 [29]	Parity of hospital order	70	70	58.5 (2.8)	57.5 (3.5)	53%	46%	C + TXLC 4 capsules each time, tid	CT + atorvastatin 1 tablet/d	3 M	①④⑤⑥⑦⑨
Wang 2018 [30]	Random number table	50	50	58.3 (2.6)	58.3 (2.6)	53%	53%	C + TXLC 2 capsules each time, tid	CT + atorvastatin 10 mg/d	1 M	①②③
Song and Ding 2012 [31]	NR	49	49	52.64 (2.36)	49.64 (3.36)	55%	49%	C + TXLC 4 capsules each time, tid	CT + atorvastatin 20 mg/d	3 M	①④⑤⑥⑦⑨
Zeng 2012 [32]	NR	42	42	53.25 (3.51)	49.03 (3.86)	60%	55%	C + TXLC 4 capsules each time, tid	CT + atorvastatin 20 mg/d	3 M	①④⑤⑥⑦

*Note.* M: month; W: week; D: day; T: treatment group; C: conventional group; CT: conventional treatment; TXLC: tongxinluo capsule; ① effectiveness of clinical treatment; ② frequency of angina pectoris; ③ duration of angina pectoris; ④ TC; ⑤ TG; ⑥ LDL-C; ⑦ HDL-C; ⑧ CRP; and ⑨ adverse reactions.

**Table 3:** Preferred Reporting Items for Systematic Reviews and Meta-Analyses (PRISMA) checklist.

No.	Section	Topic	Hang et al. 2016 [18]	Liu and An 2016 [19]	Qiao 2017 [20]	Song 2018 [21]	Nie 2018 [22]	Li 2017 [23]	Yuan 2018 [24]	Zhao et al. 2016 [25]	Qian 2014 [26]	Qiao 2017 [27]	Jiang et al. 2019 [28]	Wu 2017 [29]	Wang 2018 [30]	Song and Ding 2012 [31]	Zeng 2012 [32]
1	Title	Title	Yes	Yes	Yes	Yes	Yes	Yes	Yes	Yes	Yes	Yes	Yes	Yes	Yes	Yes	Yes

2	Abstract	Structured summary	Yes	Yes	Yes	Yes	Yes	Yes	Yes	Yes	Yes	Yes	Yes	Yes	Yes	Yes	Yes

3	Introduction	Rationale	Yes	Yes	Yes	Yes	Yes	Yes	Yes	Yes	Yes	Yes	Yes	Yes	Yes	Yes	Yes
4		Objectives	Yes	Yes	Yes	Yes	Yes	Yes	Yes	Yes	Yes	Yes	Yes	Yes	Yes	Yes	Yes

5	Methods	Protocol and registration	No	No	No	No	No	No	No	No	No	No	No	No	No	No	No
6		Eligibility criteria	Yes	Yes	Yes	Yes	Yes	Yes	Yes	Yes	Yes	Yes	Yes	Yes	Yes	Yes	Yes
7		Information sources	Yes	Yes	Yes	Yes	Yes	Yes	Yes	Yes	Yes	Yes	Yes	Yes	Yes	Yes	Yes
8		Search	Yes	Yes	Yes	Yes	Yes	Yes	Yes	Yes	Yes	Yes	Yes	Yes	Yes	Yes	Yes
9		Study selection	Yes	Yes	Yes	Yes	Yes	Yes	Yes	Yes	Yes	Yes	Yes	Yes	Yes	Yes	Yes
10		Data collection process	Yes	Yes	Yes	Yes	Yes	Yes	Yes	Yes	Yes	Yes	Yes	Yes	Yes	Yes	Yes
11		Data items	Yes	Yes	Yes	Yes	Yes	Yes	Yes	Yes	Yes	Yes	Yes	Yes	Yes	Yes	Yes
12		Risk of bias in individual studies	Yes	Yes	Yes	Yes	Yes	Yes	Yes	Yes	Yes	Yes	Yes	Yes	Yes	Yes	Yes
13		Summary measures	Yes	Yes	Yes	Yes	Yes	Yes	Yes	Yes	Yes	Yes	Yes	Yes	Yes	Yes	Yes
14		Synthesis of results	Yes	Yes	Yes	Yes	Yes	Yes	Yes	Yes	Yes	Yes	Yes	Yes	Yes	Yes	Yes
15		Risk of bias across studies	No	No	No	No	No	No	No	No	No	No	No	Yes	No	No	No
16		Additional analyses	Yes	No	No	No	Yes	Yes	Yes	Yes	Yes	Yes	Yes	No	Yes	No	No

17	Results	Study selection	Yes	Yes	Yes	Yes	Yes	Yes	Yes	Yes	Yes	Yes	Yes	Yes	Yes	Yes	Yes
18		Study characteristics	Yes	Yes	Yes	Yes	Yes	Yes	Yes	Yes	Yes	Yes	Yes	Yes	Yes	Yes	Yes
19		Risk of bias within studies	Yes	Yes	Yes	Yes	Yes	Yes	Yes	Yes	Yes	Yes	Yes	Yes	Yes	Yes	Yes
20		Results of individual studies	Yes	Yes	Yes	Yes	Yes	Yes	Yes	Yes	Yes	Yes	Yes	Yes	Yes	Yes	Yes
21		Synthesis of results	Yes	Yes	Yes	Yes	Yes	Yes	Yes	Yes	Yes	Yes	Yes	Yes	Yes	Yes	Yes
22		Risk of bias across studies	No	No	No	No	No	No	No	No	No	No	No	Yes	No	No	No
23		Additional analysis	Yes	No	No	No	Yes	Yes	Yes	Yes	Yes	Yes	Yes	No	Yes	No	No

24	Discussion	Summary of evidence	Yes	Yes	Yes	Yes	Yes	Yes	Yes	Yes	Yes	Yes	Yes	Yes	Yes	Yes	Yes
25		Limitations	Yes	Yes	Yes	Yes	Yes	Yes	Yes	Yes	Yes	Yes	Yes	Yes	Yes	Yes	Yes
26		Conclusions	Yes	Yes	Yes	Yes	Yes	Yes	Yes	Yes	Yes	Yes	Yes	Yes	Yes	Yes	Yes

27	Funding	Funding	Yes	Yes	Yes	Yes	Yes	Yes	Yes	Yes	Yes	Yes	Yes	Yes	Yes	Yes	Yes

**Table 4:** Meta-analysis results of important outcome measures.

Outcome measures	Number of included studies	Results of heterogeneity test		Effect model	Results of meta-analysis	
		*I* ^2^ (%)	*P*		95% CI	*P*
CRP	7	85	<0.00001	Random	SMD = −2.06 (−2.56, −1.57)	<0.00001
Frequency of angina pectoris	5	88	<0.00001	Random	SMD = −1.41 (−1.97, −0.85)	<0.00001
Duration of angina pectoris	4	93	<0.00001	Random	MD = −2.30 (−3.39, −1.21)	<0.0001
Adverse reactions	6	0	0.92	Fixed	RR = 0.84 (0.51, 1.39)	0.50

**Table 5:** GRADE summary table of outcome indicator evidence quality.

Tongxinluo capsule combined with atorvastatin for coronary heart disease						
Patient or population: patients with coronary heart disease						
Settings: in the hospital and home-based						
Intervention: tongxinluo capsule combined with atorvastatin						

**Outcomes**	**Illustrative comparative risks** *∗ * **(95% CI)**		**Relative effect (95% CI)**	**No. of participants (studies)**	**Quality of the evidence (GRADE)**	**Comments**
	Assumed risk	Corresponding risk				
	**Control**	**Tongxinluo capsule combined with atorvastatin**				

**Effectiveness of clinical treatment** ^**9**^	**Study population**		**RR 1.24** (1.18 to 1.29)	1,480 (14 studies)	⊕⊕⊝⊝**Low**^a,b^	
	**750 per 1000**	**930 per 1000** (885 to 967)				
	**Moderate**					
	**730 per 1000**	**905 per 1000** (861 to 942)				

**TC** ^**7**^		The mean total cholesterol in the intervention groups was **1.21 lower** (1.53 to 0.89 lower)		960 (9 studies)	⊕⊕⊕⊝**Moderate**^a^	

**TG** ^**7**^		The mean triacylglycerol in the intervention groups was **0.73 lower** (0.81 to 0.65 lower)		852 (9 studies)	⊕⊕⊝⊝**Low**^a,b^	

**HDL-C** ^**7**^		The mean high density lipoprotein cholesterol in the intervention groups was **0.27 higher** (0.23 to 0.31 higher)		643 (6 studies)	⊕⊕⊕⊝**Moderate**^a^	

**LDL-C** ^**7**^		The mean low density lipoprotein cholesterol in the intervention groups was **0.72 lower** (0.8 to 0.64 lower)		764 (8 studies)	⊕⊕⊝⊝**Low**^a,b^	

**Frequency of angina pectoris** ^**5**^		The mean frequency of angina pectoris in the intervention groups was **1.41 standard deviations lower** (1.97 to 0.85 lower)		532 (5 studies)	⊕⊝⊝⊝**Very low**^a,c,d^	

**Duration of angina pectoris** ^**5**^		The mean duration of angina pectoris in the intervention groups was **2.30 lower** (3.39 to 1.21 lower)		372 (4 studies)	⊕⊕⊝⊝**Low**^a,d^	

**CRP** ^**6**^		The mean CRP in the intervention groups was **2.06 standard deviations lower** (2.56 to 1.57 lower)		690 (7 studies)	⊕⊕⊝⊝**Low**^a,c^	SMD −2.06 (−2.56 to −1.57)

**Adverse reactions** ^**5**^	**Study population**		**RR 0.84** (0.51 to 1.39)	695 (6 studies)	⊕⊕⊝⊝**Low**^a,d^	
	**89 per 1000**	**75 per 1000** (45 to 124)				
	**Moderate**					
	**87 per 1000**	**73 per 1000** (44 to 121)				

^*∗*^The basis for the assumed risk (e.g., the median control group risk across studies) is provided in footnotes. The corresponding risk (and its 95% confidence interval) is based on the assumed risk in the comparison group and the relative effect of the intervention (and its 95% CI). CI: confidence interval; RR: risk ratio; GRADE working group grades of evidence; high quality: further research is very unlikely to change our confidence in the estimate of effect; moderate quality: further research is likely to have an important impact on our confidence in the estimate of effect and may change the estimate; low quality: further research is very likely to have an important impact on our confidence in the estimate of effect and is likely to change the estimate; very low quality: we are very uncertain about the estimate. ^a^Downgraded one level as random sequence generation, allocation concealment, blinding of participants and personnel, blinding of outcome assessment, or selective reporting were poorly described in over 50% of the included studies. ^b^Publication bias. ^c^Unit conversion may produce interstudy heterogeneity. ^d^Fewer patients and observations included in the study. ^1,2,3^Unimportant outcome indicators. ^4,5,6^Important outcome indicators. ^7,8,9^Critical outcome indicators.

## Data Availability

No data were used to support this study.

## Abbreviations

CHD: Coronary heart disease
TXLC: Tongxinluo capsule
TCM: Traditional Chinese medicine
PRISMA: Preferred reporting items for systematic reviews and meta-analyses
AMSTAR 2: Assess the methodological quality of systematic reviews
GRADE: Grading of Recommendations Assessment, Development and Evaluation
TC: Total cholesterol
TG: Triglycerides
LDL-C: Low-density lipoprotein cholesterol
HDL-C: High-density lipoprotein cholesterol
CRP: C-reactive protein
RCTs: Randomized controlled trials
RR: Risk ratio
CI: Confidence interval
MD: Mean difference
SMD: Standardized mean difference
NR: Not report
tid: Ter in die
CONSORT: Consolidated standards of reporting trials.
