# Supplementary figures and images for: Tongxinluo Capsule Combined with Atorvastatin for Coronary Heart Disease: A Systematic Review and Meta-Analysis
Source: Evid Based Complement Alternat Med. 2021 Jul 17;2021:9413704. doi: 10.1155/2021/9413704 (PMC8313336; doi:10.1155/2021/9413704)

Figure. S1: Funnel plot of effectiveness of clinical treatment


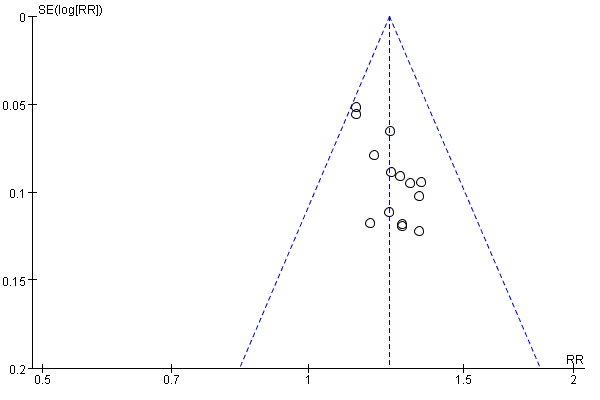


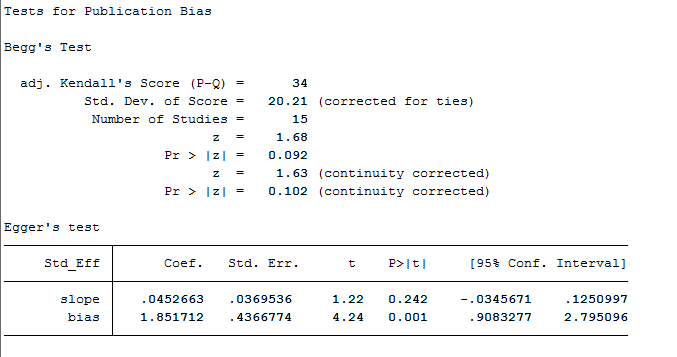
Figure. S2: Egger’s test

Supplement: Supplementary Materials — Supplementary data to this article can be found in supplementary materials. Figure S1: funnel plot of the effectiveness of clinical treatment. Figure S2: Egger's test. [file 9413704.f1.docx]
